# Comparison of the evaluation of formative assessment at two medical faculties with different conditions of undergraduate training, assessment and feedback

**DOI:** 10.3205/zma001334

**Published:** 2020-06-15

**Authors:** Katrin Schüttpelz-Brauns, Yassin Karay, Johann Arias, Kirsten Gehlhar, Michaela Zupanic

**Affiliations:** 1 Medical Faculty Mannheim at Heidelberg University, Mannheim, Germany; 2 University of Cologne, Medical Faculty, Cologne, Germany; 3 RWTH Aachen University, Medical Faculty, Aachen, Germany; 4 Carl von Ossietzky University, School of Medicine and Health Sciences, Oldenburg, Germany; 5 University Witten/Herdecke, Faculty of Health, Witten, Germany

**Keywords:** formative assessment, medical education, progress test, test effort

## Abstract

**Introduction: **Both formative and summative assessments have their place in medical curricula: formative assessment to accompany the learning process and summative assessment to ensure that minimum standards are achieved. Depending on the conditions of undergraduate training, assessment and feedback, students place more or less importance on formative assessment, and thus the fulfilment of its function may be questionable. This study describes how the low-stakes formative Berlin Progress Test (BPT) is embedded at two medical faculties with partially different framework conditions and what effects these have on the students' testing efforts and the evaluation of the test, especially the perception of its benefits and (intangible) costs, such as non-participation in contemporaneous activities and emotional impairments.

**Methods: **In this study, the proportion of non-serious BPT participants at two medical faculties (total sample: N_F1_=1,410, N_F2_=1,176) in winter term 2015/16 was determined both by the number of unanswered questions on the test itself and in a survey using a standardized instrument (N_F1_=415, N_F2_=234). Furthermore, open questions were asked in this survey about perceived benefits and perceived costs, which were analyzed with qualitative and quantitative methods.

**Results: **The BPT is generally better accepted at Faculty 2. This can be seen in the higher proportion of serious test takers, the lower perceived costs and the higher reported benefit, as well as the higher proportion of constructive comments. Faculty 2 students better understood the principle of formative testing and used the results of the BPT as feedback on their own knowledge progress, motivation to learn and reduction of exam fear.

**Discussion:** When medical faculties integrate formative assessments into the curriculum, they have to provide a framework in which these assessments are perceived as an important part of the curriculum. Otherwise, it is questionable whether they can fulfil their function of accompanying the learning process.

## Zusammenfassung

**Einführung: **Sowohl formative als auch summative Prüfungen haben ihre Berechtigung in medizinischen Curricula: formative Prüfungen zur Begleitung des Lernprozesses und summative Prüfungen zur Sicherung des Erreichens von Mindeststandards. Je nach Studien-, Prüfungs- und Feedbackbedingungen wird formativen Prüfungen durch die Studierenden mehr oder weniger Bedeutung beigemessen und entsprechend kann die Erfüllung ihrer Funktion fraglich sein. In dieser Studie wird beschrieben, wie der nicht-bestehensrelevante formative Progress Test Medizin (PTM) an zwei Medizinischen Fakultäten mit partiell unterschiedlichen Rahmenbedingungen eingebettet ist und welche Auswirkungen diese auf das Testbemühen der Studierenden und die Bewertung des Tests, insbesondere der Wahrnehmung von dessen Nutzen und (immateriellen) Kosten, wie Nicht-Teilnahme an zeitgleichen Angeboten oder emotionale Beeinträchtigungen, haben.

**Methoden: **In dieser Studie wurde der Anteil der nicht ernsthaften PTM-Teilnehmenden an zwei Medizinischen Fakultäten (Gesamtstichproben: N_F1_=1.410, N_F2_=1.176) im WS 15/16 sowohl durch die Zahl nicht beantworteter Fragen im Test selbst als auch im Rahmen einer Befragung mit einem standardisierten Instrument (N_F1_=415, N_F2_=234) bestimmt. Weiterhin wurden in dieser Befragung offene Fragen zum wahrgenommenen Nutzen bzw. den wahrgenommenen Kosten gestellt, welche mit qualitativen und quantitativen Methoden ausgewertet wurden.

**Ergebnisse: **Der PTM wird an Fakultät 2 insgesamt besser angenommen. Dies zeigt sich in dem höheren Anteil ernsthafter Testteilnehmenden, den niedrigeren wahrgenommenen Kosten und dem höheren berichteten Nutzen sowie dem größeren Anteil an konstruktiven Kommentaren. Studierende der Fakultät 2 haben das Prinzip des formativen Prüfens besser verstanden und nutzen die Ergebnisse des PTM als Feedback über den eigenen Wissensfortschritt, zur Lernmotivation und zur Reduktion von Prüfungsangst.

**Diskussion: **Wenn Medizinische Fakultäten formative Prüfungen in das Curriculum integrieren, müssen sie Rahmenbedingungen schaffen, in denen diese Prüfungen als wichtiger Teil des Curriculums wahrgenommen werden. Ansonsten ist es fraglich, ob sie ihrer Funktion der Begleitung des Lernprozesses gerecht werden können.

## Introduction

According to the Medical Licensing Regulations (ÄAppO), §2 subsection 7, successful participation in the pre-clinical phase must be proven with 17 major course assessments (Appendix 2a) and in the clinical phase with 40 major course assessments (Appendix 2b). This proof is either provided by a graded assessment, which tests the learning outcome of a section, such as a subject or module, or by a pass/fail assessment. Therefore, these are assessments of learning or summative assessment [1]. On the other hand, there are assessments that accompany the learning process. These formative assessments [1] promote continuous and in-depth learning [2]. Feedback is a central aspect of continuous learning in that gaps in learning are identified and corrected in a targeted manner. Continuous learning prepares for lifelong learning, which is becoming increasingly important due to the fast pace of knowledge and constantly changing requirements [3]. There are already some studies on formative assessment that investigate the effect on learning. This so-called educational impact is part of the model of utility of assessment methods [4] and can be seen as an indication that the effect on continuous learning is given by the formative assessment or its feedback. Wade et al. developed a questionnaire to compare the perception of progress tests – a type of formative assessment (see below) – as a learning tool at two different medical schools and found that the learning environment has an impact on the appreciation of progress tests as a learning support [5]. Cobb et al. asked students in semi-qualitative interviews about their perception of DOPS (formative direct observation of procedural skills) compared to MCQs (summative multiple-choice assessment) and found that formative testing promoted deeper learning, but summative testing was more important for students [6]. In a questionnaire study at the Faculty of Health Sciences in Maastricht, students found summative block tests more rewarding and did not use the results of the progress test for self-regulated learning [7]. Embedding feedback through/with progress tests in a comprehensive examination programme increased student use of the progress test feedback tool and integration into learning [8] . 

Both the continuous accompaniment of the learning process through formative assessment and the assurance of the achievement of minimum standards in the form of summative assessment are justified in the medical curriculum. 

It can be assumed that the two aims – learning for the assessment vs. assessment for learning – are pursued by students with varying degrees of intensity, which can be seen in the different extent of test effort. This can be explained with the Expectancy Value Theory of Wigfield & Eccles [9]. The Expectancy Value Theory states that the motivation to complete a task depends on two components: the expectation of being able to solve a task and the value that this task has for the individual. Wigfield & Eccles [9] distinguish four different components that can make up this value:

- Performance value (to master the task in the best possible way),
- Intrinsic value (the fun or joy in solving the task),
- Utility value (how well the task fits into future plans, i.e. how useful the task is)
- Costs (costs in the strict sense, the extent to which activities compete with each other, but also emotional costs).

With regard to summative and formative assessment, there is a difference in the value given to a task, the assessment. The value that a task or assessment has for each student is also influenced by the general conditions at the medical school. This has a decisive influence on the perceived benefits and costs. For example, it is very likely that the summative assessments are in the focus of the students, if the evidence required by the ÄAppO has to be proven at their medical faculty exclusively by summative assessment. In the worst case, they learn extremely efficiently, i.e. they learn all the required content shortly before the corresponding assessment, the so-called bulimia learning [10]. From the students' point of view, the benefit – passing the assessment – would be maximum at minimum cost. The knowledge acquired in this way runs the risk of being “ticked off” after the exam and soon forgotten [11].

Especially with regard to the benefits and costs of Expectancy Value Theory, faculties can provide framework conditions to increase the motivation to use formative assessment and thus the influence on learning. Formative assessment can be seen as an additional effort, especially if summative assessments and/or work-intensive courses (study load) have to be taken in parallel. If, on the other hand, formative assessment is perceived as a meaningful and valuable component of the overall curriculum and is valued by faculty members, the benefit of formative assessment could be regarded as high – despite contemporaneously graded assessment and high study load. 

The formative progress tests in medicine offer an opportunity to investigate under which conditions formative assessment can be successfully implemented despite the competing summative assessments that are perceived as more useful. 

Progress tests are multiple-choice tests that regularly test students’ medical knowledge during undergraduate training at the level of a new graduate and compare it with the knowledge level of fellow students in the same semester in order to identify gaps in the current level of knowledge and to constructively influence learning behaviour. All types of progress tests provide feedback, but are used differently with regard to their stakes. In the Dutch consortium and in the USA, for example, the results of the progress test are accumulated over several test times for each individual [12], [13]. This means that the progress tests are not graded, but they do have an influence on the progress of study. In Germany and Austria, participation is mandatory, but is not graded (low stakes) [2], [14]. In the German-speaking Progress Test Medicine (BPT) consortium, test preparation and analyses are carried out centrally at the Charité University Medicine in Berlin. All test takers receive detailed feedback from Berlin about 4-6 weeks after the test on their results over the years, but also in comparison with their fellow students, differentiated by organ systems and subjects. The varying degree of test efforts at the individual faculties is reflected in the proportion of serious test takers, which is routinely computed after each test. In the case of the low-stakes BPT, it is shown that there are very different proportions of serious test takers at the various faculties. Proportions of 75-90% were reported by the participating faculties [15].

This study examines how the low-stakes BPT is embedded in two faculties and how this affects the students’ testing efforts and the perception of the progress test, especially the perception of costs and benefits as a formative test. The framework conditions for the BPT differ at both faculties, among other things, in their integration into each curriculum: The conditions of undergraduate training, assessment and feedback are shown in detail in table 1 (Tab. 1). 

Looking at the conditions of assessment and feedback at both faculties, the proportion of serious test takers and the associated perception of the costs and benefits of the BPT should be comparable, as both faculties have conditions that should have a positive effect on motivation and, accordingly, on testing efforts. 

Students at Faculty 1 have a choice that is not available at Faculty 2. They can choose which 8 out of 10 BPTs they would like to take. According to the Self-Determination Theory of Ryan & Deci [16], this should increase intrinsic motivation and thus increase the proportion of serious test takers. 

In addition, Faculty 1 provides immediate feedback from the computer-based administration. Immediate feedback is important for completing tasks and being satisfied with the work [17], [18], [19]. Therefore, the condition of computer-based administration should also increase the test effort and thus the proportion of serious test takers. 

Although the feedback is immediate, there is no dialogue about the results at Faculty 1. The dialogue about the results is integrated into the mentoring programme at Faculty 2. Dialogue is essential for effective feedback and thus for the functioning of formative assessment [20], [21], [22]. This should increase the* perceived benefit* of the BPT at Faculty 2.

Since the BPT is communicated as an assessment at Faculty 2, as opposed to Faculty 1, where it is presented as an evaluation, the BPT at Faculty 2 should be perceived as more useful for another reason. As Heeneman et al. were able to show in their study, students use the feedback system of the moderate-stakes progress test more and have higher test scores when the progress test is integrated into a holistic examination system [8]. The higher test scores were seen as an indirect indicator of test efforts. At the same time, the *perceived costs* are lower when the formative test is part of the assessment system. 

Taking into account the conditions at the two faculties and their theoretical influences on the test effort, measured by the proportion of serious vs. non-serious test takers, and on the perceived costs and benefits of the BPT, the following hypotheses can be derived:

1. The proportion of non-serious test takers at Faculty 1 (F1) is lower than at Faculty 2 (F2).
2. The perceived costs of the BPT are higher at Faculty 1 (F1) than at Faculty 2 (F2).
3. The perceived benefit of the BPT is lower at Faculty 1 (F1) than at Faculty 2 (F2).

## Methods

The study is conducted as a mixed-method approach, in which the proportions of non-serious test takers are determined quantitatively. In the qualitative part, the themes are identified which are relevant for the students in terms of perceived benefits and costs in relation to the BPT at both faculties. 

### Sample 

In winter semester 2015/16, N=1,410 (F1) and N=1,176 (F2) medical students participated in the BPT. This corresponds to 50% of the enrolled medical students at F1 and 61% at F2. The proportion of female students at the faculties is 62% (F1) and 68% (F2).

#### Material 

The proportion of non-serious test takers was determined in two different ways. On the one hand, those students who chose the “don't know” option for all questions or skipped all questions when filling out the test in winter term 2015/16 were identified as non-serious, since even in the first semester at least two questions can be answered. On the other hand, the test effort was determined by means of the Test-Effort Short Scale (TESS) [23]. TESS consists of three five-stage Likert items with the gradations 1 to 5, which ask for the performance value (“I would like to achieve the best possible result on the BPT”), the utility value (“I find the BPT useful”) and the perceived costs (“The BPT is a valuable part of my undergraduate training”). The mean value is calculated from the answers to all three questions. Students who did not agree with these statements and answered all questions with 1 (corresponding to a TESS score of 1) are categorized as non-serious test takers. Both procedures each have a methodological disadvantage that could reduce their validity. The disadvantage of self-response tests is that there is an unknown percentage of students who answer in a socially desirable manner. This means that they could indicate a higher level of testing effort than is actually the case. The disadvantage of identification via the “don't know” option is that there may also be so-called pattern markers. These are test takers who answer all questions but do so without knowing the text of the questions [24]. Due to these disadvantages, both methods have been used in parallel. 

In order to make the perceived costs and benefits measurable, we have asked open questions. Both the concept of costs and the concept of benefits are very abstract. Therefore, we asked formally balanced questions that provoke possible answers that can be assigned to these two terms. These are, on the one hand, questions about the disadvantages and advantages of the BPT, but also questions directly about the benefits of the BPT. Students who use the BPT should also talk to other people about their results, such as their mentor, in order to change their own learning behaviour. 

The perceived costs were addressed in two open questions:

- Do you feel emotionally impaired by the BPT? (Question 1)
- What disadvantages do you see in the BPT? (Question 2)

The perceived benefit was determined by means of five questions (two closed and three open questions) on different aspects:

- Dialogue with other people about the results of the BPT with the sub-questions:
  - I talk with fellow students about my results on the BPT. (Likert item, with 1 “does not apply” to 5 “applies”);
  - I talk to my mentor about my results on the BPT. (Likert item, with 1 “does not apply” to 5 “applies”);
  - I talk to other people about my results on the BPT. With ... (open question, Question 3).
- Do you use the results of the BPT for other purposes? (open question, Question 4)
- What advantages do you see in the BPT? (open question, Question 5)

There was no limit to the number of comments that students could make on the open questions. 

In addition, the questionnaire asked for gender and semester of study in order to check the comparability of both groups. 

#### Procedures

At Faculties 1 and 2, the BPT took place in the first weeks of the semester on the university premises and under supervision. At least two non-overlapping dates were planned for each cohort, which the students could choose independently. At both faculties the testing was computer-based. At Faculty 2, additional dates for paper-based testing were offered.

The students at both faculties participated regularly in the BPT. At the beginning of the test, students were informed about the overall study in addition to the regular introduction. The overall study examines the motivation on the BPT and its influence on learning on the BPT. Therefore, the questionnaire contained more questions than the ones given here. In the regular introduction, the participants were asked to complete the questionnaire after the test had been completed and were informed that this participation was voluntary and anonymous. The Ethical Review Board of the Medical Faculty Mannheim, Heidelberg University, approved the study (2015-542-N-MA).

#### Analyses

The proportion of non-serious test takers per faculty was checked for independence in each case using a χ^2^ test. Since the sample is very large and therefore even small differences can become significant, the effect size was measured with Cohen’s w for contingency tables and Cohen’s d for metric data (see below) in order to assess the relevance of differences [25]. The effect size w is categorized as no effect with w<0.1, small effect with w<0.3, moderate effect with w<0.5 and large effect with w≥0.5 [25]. 

To compare the TESS scores between the two faculties, a t-test for independent samples with unequal variances was calculated, and the effect size d according to Cohen [25], with pooled standard deviations according to Leonhart (2004) was calculated [26]. The categorization of d is as follows: d<0.2 no effect, d<0.5 small effect, d<0.8 moderate effect and d≥0.8 large effect. 

The analyses of the two Likert items (*“I talk with fellow students”* and *“I talk with my mentor about my results on the BPT”*) were recoded so that statements of 4 or 5 were considered as agreement. 

Qualitative and quantitative methods were used to evaluate the open questions about the costs and benefits of the BPT. The data from the evaluation questionnaire were analysed in three steps: First, two authors (KG, MZ) examined all comments on the open questions and coded them independently of each other using the thematic content analysis [27]. In a second step, after joint discussion of discrepancies and new perspectives, these codes were again independently grouped into categories and a category list was created. In the third step, this category list was checked for inter-coder reliability with perfect matches (100% each) for the open questions 1 (8 categories), 3 (7 categories) and 4 (4 categories). Very good matches were found for the open questions 2 (94%, 9 categories) and question 5 (97%, 12 categories), so that this category list was used in the further analyses. The number of entries per category is given in the results section. The corresponding percentages refer to the total number of mentions for the given question. 

## Results

### Descriptives

415 students at F1 and 453 students at F2 took part in the survey. 234 students at F1 answered the questions included in the analysis (57% female, respondents=56% of the sample, 234/415). At F2, 248 students answered these questions (71% female; respondents=55% of the sample, 248/453). An overview can be found in table 2 (Tab. 2). 

The two universities differed in a statistically significant manner in the distribution of the sexes (χ^2^=10.52, df=1, p<.001) with a higher proportion of women at F2, but not in the distribution of students in the pre-clinical and clinical phase of their undergraduate training (n. s.). No statistically significant effects were found in preliminary analyses, so that the variable sex was not included as a covariate in the evaluations. 

#### Proportion of non-serious test takers on the BPT 

Regardless of the approach to operationalisation, it is shown that at Faculty 1 the proportion of non-serious test takers is significantly higher than at Faculty 2.

At F1 there are N_F1_=173/1,410 (12%) students who answered all questions on the BPT with “don't know” or not at all, at F2 there are N_F2_=5/1,191 (<1%). This is a significant difference with χ^2^(1)=142.20; p<0.001 and a small effect of w=0.23. 

On the questionnaire, the following average TESS values, which reflect the self-evaluated test effort, were calculated at F1 for 291/415 (70%) students: M_F1_=2.51; SD_F1_=1.08 and at F2 of 409/453 (90%) students M_F2_=3.63, SD_F2_=0.88. This difference is also significant with T(543.80)=14.68; p<0.001 and has a large effect of d=-1.19. The testing effort of the students at F2 was therefore significantly greater than at F1. If the test takers are categorized as serious vs. not serious, there are N_F1_=52/415 (13%) and N_F2_=3/453 (<1%) non-serious test takers. This difference is also significant with χ^2^(1)=68.96; p<0.001 and a moderate effect (w=0.31). 

#### Perceived costs at faculties with different examination and feedback conditions 

Overall, the students from F1 reported more frequently on perceived costs of the BPT. F2 received more positive, constructive comments than F1. Multiple answers were possible when answering Question 1: *“Do you feel emotionally impaired by the BPT?”* At F1 there were 55 responses (24% of the 234 respondents) to this question, of which 53% (29/55) were constructive. Of the 19 mentions (8% of the 248 respondents) at F2 who answered this question, 15/19 (79%) were constructive. Table 3 (Tab. 3) shows the allocation of responses to the individual categories per faculty. 

Question 2 *“What disadvantages do you see in the BPT?”* resulted in 241 responses at F1 (103% of the 234 respondents; viz. some multiple responses), 43% of which (104/241) were constructive responses. At F2, 65/105 (62%) of 105 responses (42% of the 248 responders) were constructive. The allocation of responses to the individual categories per faculty is shown in table 4 (Tab. 4). 

#### Perceived benefit at faculties with different examination and feedback conditions 

163 (39%) of the students from F1 and 309 (68%) of the students from F2 talk to other people about their BPT results. 84 (20%) of the students from F1 agreed with the statement that they talked with their fellow students about their BPT results. At F2 this number was 147 (32%). The statement that they talked with their mentor about their own results on the BPT was agreed with by 4 (1%) of the F1 students and 16 (4%) of the F2 students. A total of 75 (18%) of the participating F1 medical students and 146 (32%) of the F2 students talked with others about their BPT results. The frequency of agreement on the two closed questions, as well as the allocation of mentions to the individual categories per faculty for the other persons (open Question 3), are listed in table 5 (Tab. 5). 

Question 4 *“Do you use the results of the BPT for other purposes? If so, how?”* There were 72/234 responses from F1 (31% of respondents) and 33/248 responses from F2 (13% of respondents). Although there were more responses at F1 than at F2, a high percentage of the responses from F1 were more likely to be in categories with negative connotations (70/72 responses, 97%), compared to only 22/33 responses (67%) from F2 with more negative connotations, as documented in table 6 (Tab. 6). 

In response to Question 5 *“What advantages do you see in the BPT?”* there were just over 200 responses from both faculties (F1 with 207/234, 88% and F2 with 202/248, 81% of the respondents). At F1 163/234 (79%) of the responses could be assigned to positive categories, at F2 198/248 (98%), as shown in table 7 (Tab. 7). 

## Discussion

Formative assessment is important as an essential part of the assessment for learning. If formative assessment is not graded, it may be perceived by students as having high costs and/or lower benefits compared to summative assessment. In these cases, the proportion of non-serious test takers may be high. The present study investigated whether different framework conditions at two faculties have an influence on the test effort and the perceived costs and benefits of a formative assessment – the Berlin Progress Test (BPT). The different framework conditions can be found in the required number of participations in the BPT during undergraduate training, the presentation of the BPT, the feedback on the results, as well as the university’s implementation. Although both medical faculties are implementing measures to increase the acceptance of the BPT in order to increase test effort, the BPT is better accepted by students at Faculty 2 than at Faculty 1, as evidenced by the higher proportion of serious test takers, the lower perceived costs and higher reported benefits, and the greater proportion of constructive comments. 

### Serious test taking

The hypothesis *“The proportion of non-serious test takers at Faculty 1 is lower than at Faculty 2”* could not be confirmed. Contrary to this hypothesis, the proportion of serious test takers at Faculty 1 is lower than at Faculty 2, despite more choices and immediate feedback. Although it has been shown elsewhere that the proportion of serious respondents is higher in computer-based administration than in paper-based administration [28], several studies have already shown that several factors influence test effort. Therefore, unicentric studies can only make a marginal contribution to the explanation of the multifactorial conditions for the test effort on formative tests. 

#### Costs 

The present study was able to confirm the hypothesis “The* perceived costs of the BPT are higher at Faculty 1 than at Faculty 2.”*. The comments of the participants reflect findings from the literature that the costs of the BPT are perceived as high if the students estimate that they cannot simultaneously perform higher rated alternatives, such as learning for “real” assessment or if they feel emotional stress when filling out the test [29]. 

#### Benefits

The results for testing the hypothesis “*The perceived benefit of the BPT is lower at Faculty 1 than at Faculty 2”* must be considered in a more differentiated way. Although more students at Faculty 2 talk about their BPT results, half of those people are from outside the faculty. This is surprising because the BPT should be part of the undergraduate training and therefore students would be expected to talk mainly with their fellow students and mentors about their results. However, a mentor was rarely mentioned in answering this question, although a mentoring programme is available at F2. When asked whether students use the BPT results for other purposes, the proportion of comments made by students at Faculty 1 was higher than that of students at Faculty 2. However, this is a very high proportion of comments with negative connotations or comments that show that the results are not used for other purposes. Faculty 2 students have a better understanding of the principle behind formative testing and use the BPT results as feedback on their own knowledge progress, motivation to learn and reduction of exam fear. Although the attitude towards the BPT is more positive at Faculty 2, students at both faculties rarely mentioned that they use the BPT as a learning tool (10 mentions out of a total of 482 students who completed the questionnaire). The effect on learning is therefore questionable. However, this would be a quality criterion for the utility of an assessment method [4], especially in formative assessment where the function of the assessment is to stimulate and provide feedback on learning. The learning effect must be investigated more closely in further studies, especially since the effect on learning is questionable even in the case of moderate-stakes progress tests. Only a moderate role of the progress test in identifying strengths and weaknesses could be identified [30]. Aarts et al. showed that a majority of students used the results of the moderate-stakes progress test to monitor their knowledge, but it was not clear whether this also had a direct influence on learning [31]. This was also shown by Given et al. They found in semi-structured interviews that, although the students felt informed about their strengths and weaknesses, the feedback had no influence on future learning [32]. Yielder et al. also found with focus groups that in younger students, future learning is influenced by the progress test, but not by the feedback, rather by the content of the test [33]. Students in advanced semesters are more likely to use the progress test as a reminder that they need to learn at all. The proportion of comments on the benefits of the BPT in the present study is roughly comparable at both faculties, but it is also apparent that students at Faculty 2 are more positive about the BPT. It can therefore be concluded that the hypothesis on the perceived benefit of the BPT can be confirmed, but to limit this, its effect as a learning instrument is also questionable at Faculty 2.

#### Strengths and weaknesses

In the present study it could be shown that different conditions of assessment and feedback can be associated with different proportions of serious test takers and thus with an increased variance in test efforts. It also showed that the costs and benefits of the progress test are perceived differently at the two faculties. Faculty 2 not only had more serious test takers, but the BPT was also perceived more positively in terms of costs and benefits than at Faculty 1. 

The advantage of the present study is the direct comparison of two medical faculties where the BPT was introduced at the same time more than 15 years ago. The conditions at both faculties are comparable in many respects: both have a model study programme and three licensing state examinations, which can have an influence on the BPT results [34]. Both faculties have comparable implementation conditions for the BPT, such as the same test, mandatory participation and no admission to further courses if the BPT is not taken. On the other hand, the two conditions for the implementation of the BPT differ in their different integration into the quality management system vs. into the assessment system and in the feedback (immediate feedback of results in the case of computer-based testing vs. comparison with the solution booklet on request). In addition to the comparable conditions at the two faculties, the present study offers the mixed-method approach as a further methodological advantage, which allows both quantitative and qualitative analyses. Thus a better insight into the perception of the BPT at the two faculties was gained and it could also be shown quantitatively that the percentage of serious test takers differs greatly between the two faculties. 

The methods used to determine the proportion of serious and non-serious test takers each have limitations in their validity, such as an unknown degree of sensitivity/specificity (“objective criteria”) and the questionable significance of the self-reports (TESS score). In order to increase the validity of the results, triangulation was used to measure the test effort with different methods. Since both methods lead to the same conclusion, it can be assumed that the test effort is higher at Faculty 2 than at Faculty 1. Furthermore, the answers from the open questions also allow this conclusion to be drawn, since more constructive answers were given at Faculty 2 and also a higher benefit and lower costs were reported. According to the Expectancy Value Theory, the motivation to complete this task, meaning the test effort on the BPT, should therefore be higher at Faculty 2 than at Faculty 1.

## Conclusion

The formative BPT as an assessment for learning is intended to give students feedback on the amount of their own medical knowledge, compared to the level at which they will graduate and compared to fellow students of the same level of undergraduate training, in order to accompany and modulate the learning process in the context of continuous learning. It is intended to be an antithesis to bulimic learning, which can occur more frequently due to too many summative assessments [2]. As with other low-stakes tests, there are large variances in test effort on the BPT and thus a questionable effect on learning. It can be assumed that measures to reduce the perceived costs and increase the perceived benefit can positively influence test effort and, in the long term, the effect on learning. Even if there is presumably no problem with the test effort on moderate-stakes progress tests, studies show the limited impact on learning. Therefore, framework conditions should be identified which positively influence the perceived costs and benefits of formative assessment and thus have a long-term effect on the learning process. Since the BPT provides data for feedback on the student's knowledge status as well as the learning progress, but the use of the BPT as a learning tool is up to the students, the BPT and the use of the results for their own learning should be embedded in the curriculum. This can be done by embedding the BPT in the assessment system, both as part of the assessment regulations and in the presentation of information and results, as at Faculty 2. Further possibilities for influencing perceived costs and benefits at a faculty would be to avoid contemporaneous summative assessment during formative assessment phases [6], [8], integration in the mentoring system for all students and not only as identification for the necessary support of underachieving students [13], [30], [31], [35], [36], [37]. It would also be conceivable to use formative assessment to develop and follow up learning plans together with the mentor [38]. If formative assessment is used to provide continuous feedback on knowledge, discussed with the mentor and serves to orient future learning, as envisaged in the programmatic assessment [8], [39], then it will serve its purpose. And only then students will see the value of formative assessment. 

Although formative assessment is becoming increasingly important, it is not enough to introduce it as an add-on to the curriculum. Rather, new assessment formats also require the appropriate framework conditions to achieve the desired effect. In formative assessment, therefore, conditions must be created in which the results have a value, both as a guide through the undergraduate training and as guidance for learning behaviour. Only if equal importance is attached to formative and summative assessment will the perceived costs and benefits be comparable along with the test effort. Thus, the focus of students can be shifted to continuous learning, away from bulimic learning, because it can be assumed that students who focus their actions on merely passing MC exams will not be able to recognize the value of formative assessment at all. 

Low-stakes assessment is a good way to learn under what conditions assessment for learning works and how it can be effectively embedded in existing curricula. Therefore, further studies should investigate the extent of the individual measures and their interaction. This is a great challenge since the investigation of real conditions in medical education is made difficult by many, often uncontrollable conditions [40].

## Competing interests

The authors declare that they have no competing interests. 

## Figures and Tables

**Table 1:**
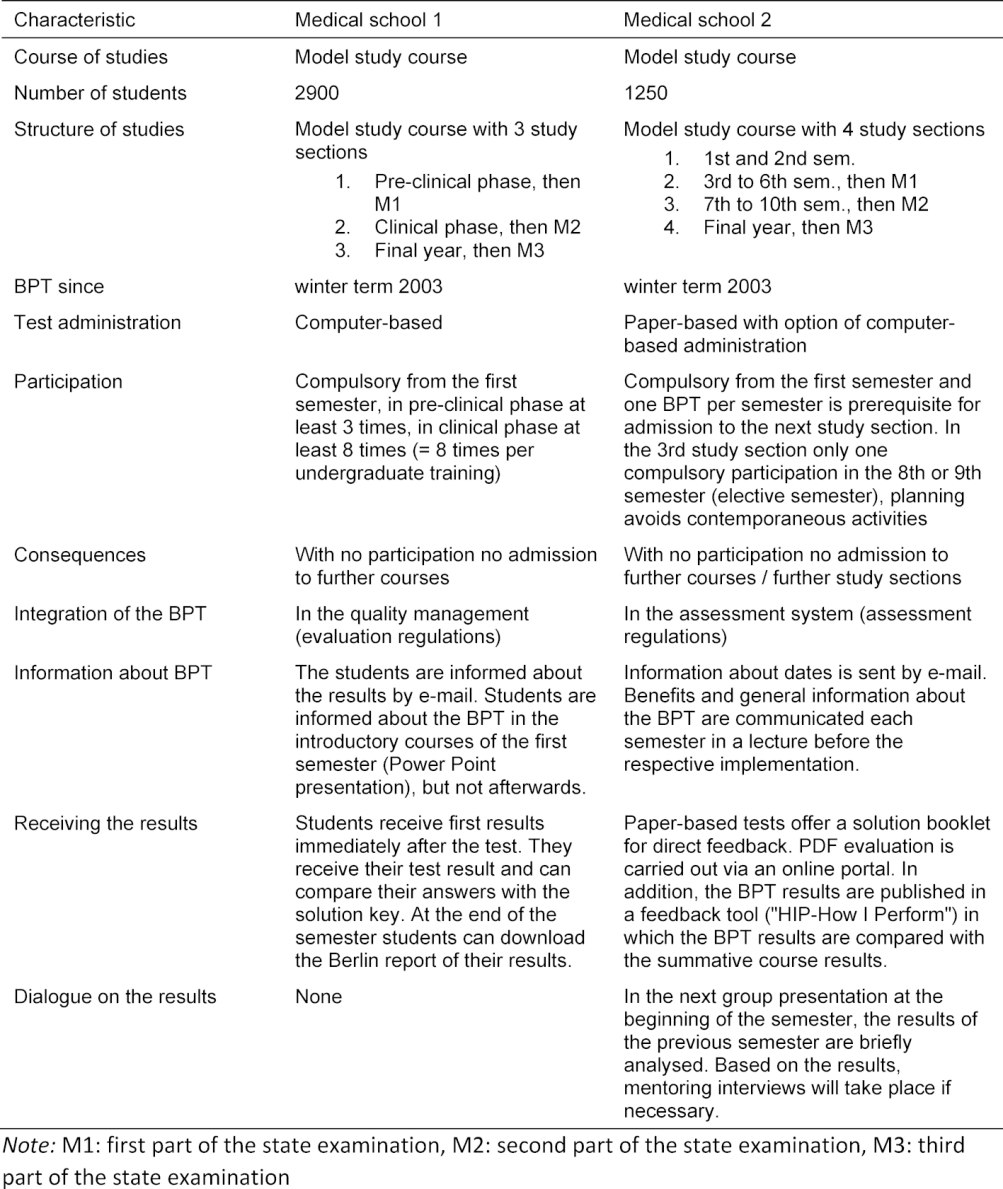
Conditions of undergraduate training, assessment and feedback at the two faculties

**Table 2:**
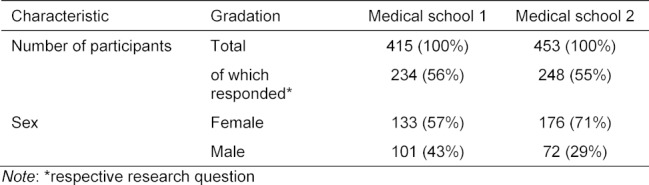
Participants in the BPT survey

**Table 3:**
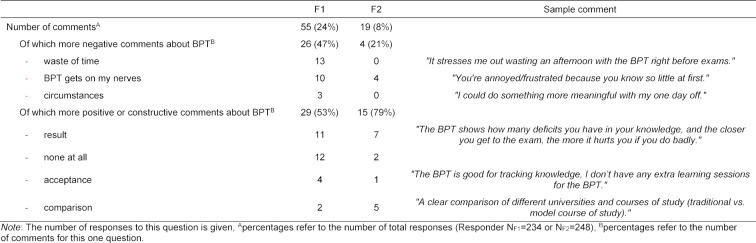
Categorisation of the responses to the open question “Do you feel emotionally restricted by the BPT? In what way?”

**Table 4:**
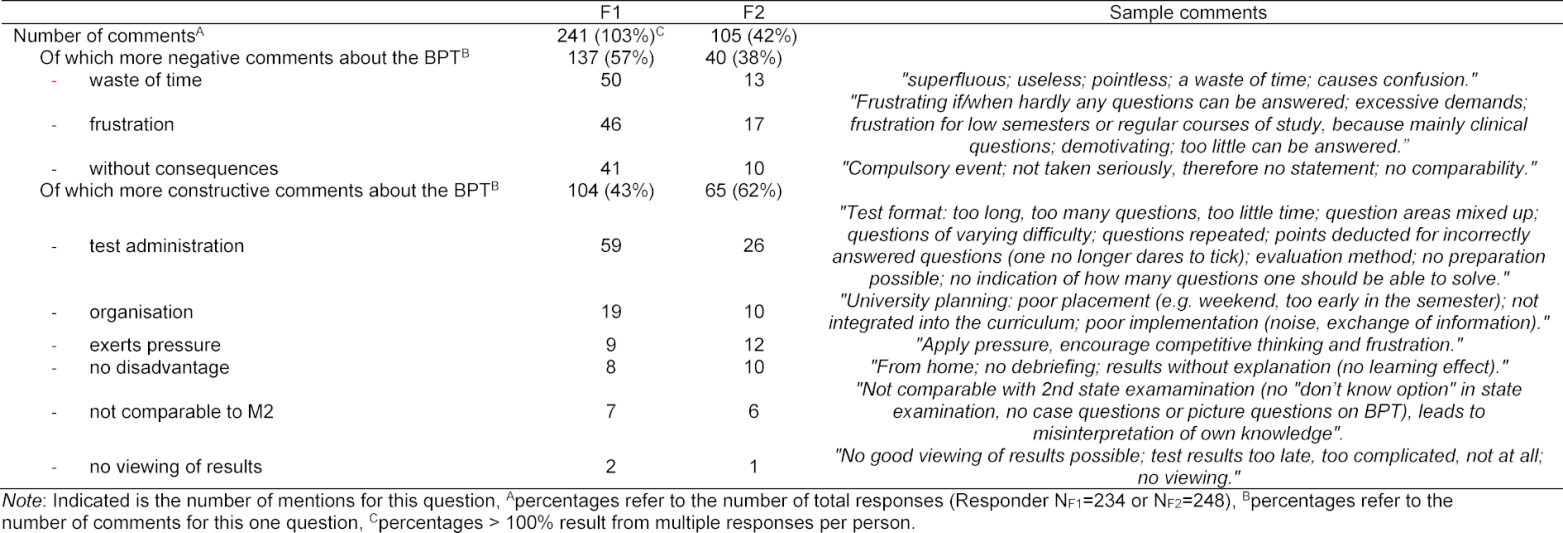
Categorisation of the responses to the open question “What disadvantages do you see in the BPT?”

**Table 5:**
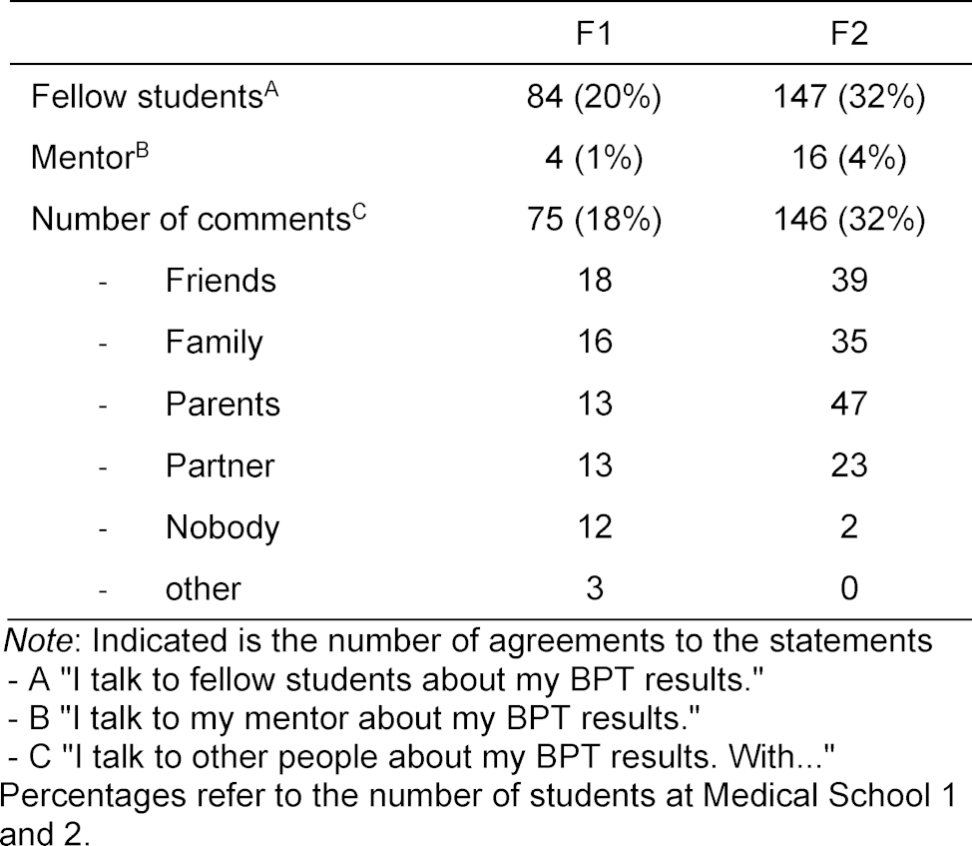
Dialogue with other people about the results of the BPT

**Table 6:**
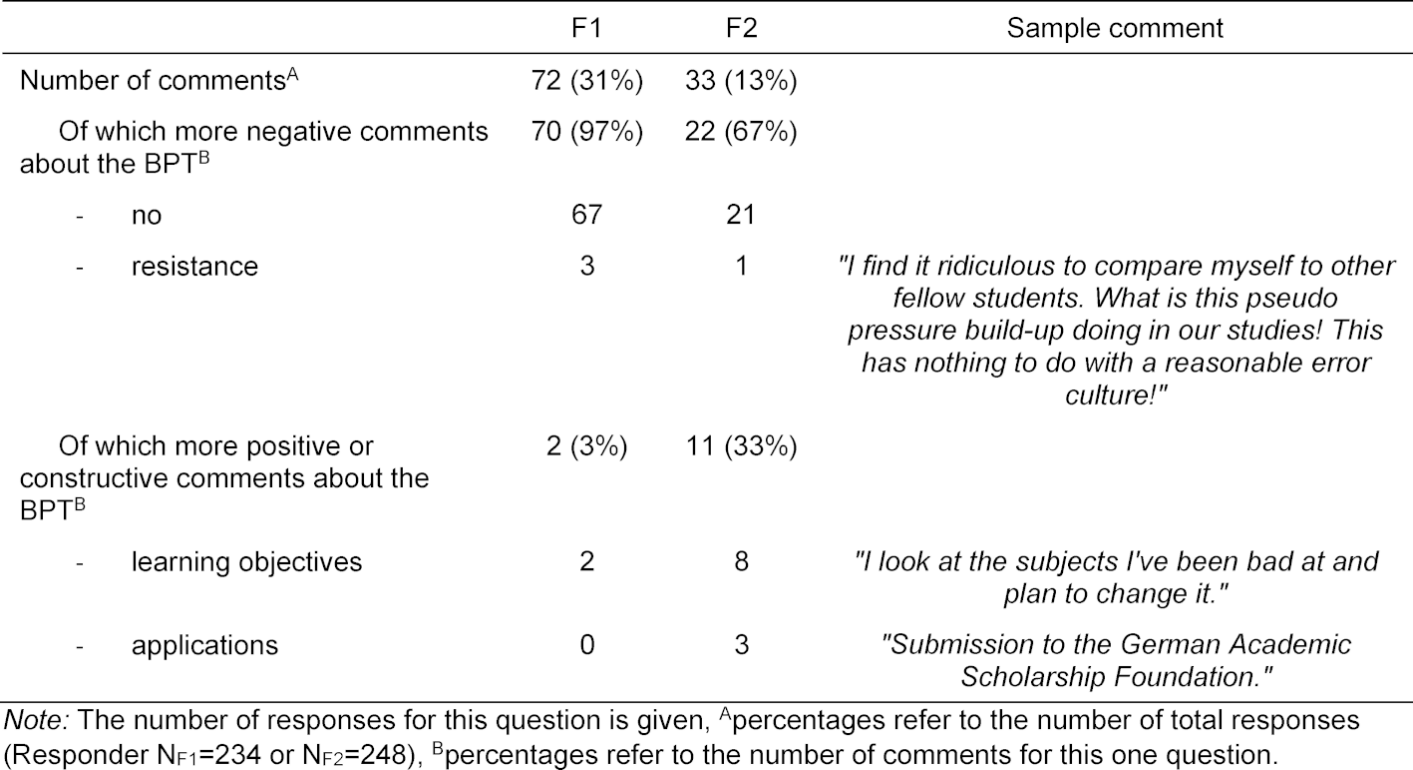
Categorisation of the responses to the open question “Do you use the results of the BPT for other purposes? If so, how?”

**Table 7:**
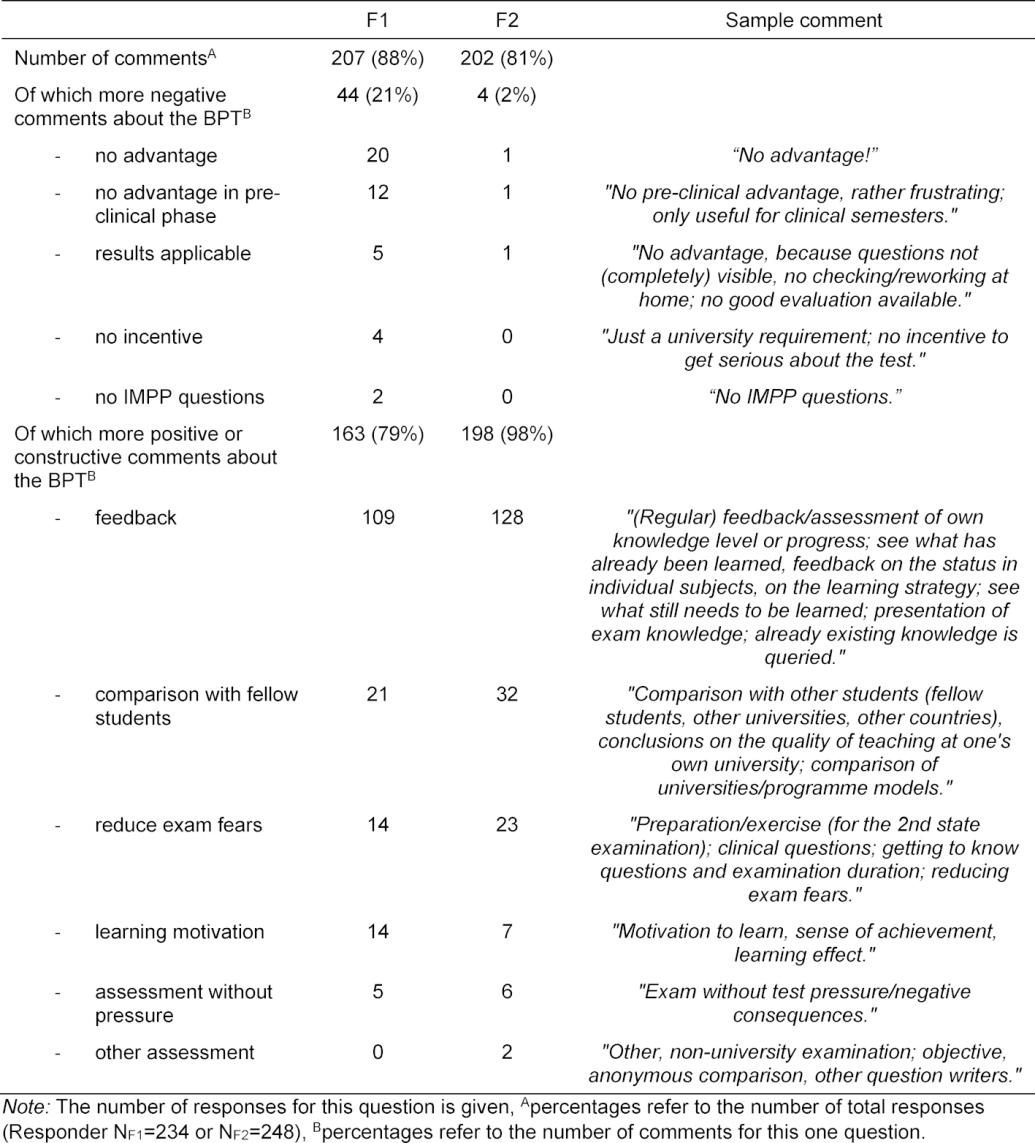
Categorisation of the responses to the open question “What advantages do you see in the BPT?”
